# Cystic fibrosis transmembrane conductance regulator modulator therapy and lived experiences in South Africa: A mixed-methods study

**DOI:** 10.4102/sajp.v82i1.2422

**Published:** 2026-08-17

**Authors:** Brenda M. Morrow, Janine Verstraete, Sinead Thistlewhite, Carese Abrahams, Teboho N. Nkosi, Tyra Donnelly, Dalziel Kennedy, Marco Zampoli

**Affiliations:** 1 Department of Paediatrics and Child Health, Faculty of Health Sciences, University of Cape Town, Cape Town, South Africa; 2 Department of Health and Rehabilitation Sciences, Faculty of Health Sciences, University of Cape Town, Cape Town, South Africa

**Keywords:** cystic fibrosis, CFTR modulator therapy, treatment burden, quality of life, participation, health equity, South Africa, mixed methods

## Abstract

**Background:**

Cystic fibrosis transmembrane conductance regulator modulator (CFTRm) therapy has transformed cystic fibrosis (CF) outcomes in high-income settings, yet access remains severely limited in South Africa. Patient-reported outcomes of CFTRm are underexplored.

**Objectives:**

To describe the lived experiences of South African people living with cystic fibrosis (PLwCF) following CFTRm initiation, focusing on respiratory symptoms, treatment burden, physical activity, social and recreational participation, and well-being.

**Method:**

A convergent mixed-methods design incorporated an online survey (*n* = 37; 48% response rate) and semi-structured interviews (*n* = 6) with PLwCF aged > 6 years. Chi-square and Fisher’s exact tests compared pre- and post-CFTRm responses; qualitative data underwent inductive thematic analysis.

**Results:**

Participants reported dramatic improvements from pre- to post-CFTRm: daily distressing respiratory symptoms decreased from 73% to 6% (*p* < 0.0001); perceived therapy burden shifted from predominantly high (78%) to predominantly low burden (87%), with a similar decline in duration of respiratory treatment: before CFTRm, 63% of participants spent > 1 h per day on respiratory treatment compared to 14% after CFTRm (*p* < 0.0001); regular exercise increased from 62% to 92% (*p* = 0.04); frequent social participation from 22% to 49% (*p* = 0.006); and perceived excellent health increased from 5% to 65% (*p* < 0.0001). Ten qualitative themes emerged: (1) respiratory health transformation, (2) reduced treatment burden, (3) normalisation of daily life, (4) enhanced physical capacity and participation, (5) profound psychosocial and (6) family impact, (7) improved nutrition, (8) reduced hospitalisations, (9) improved energy, and (10) altruistic hope for universal access.

**Conclusion:**

Cystic fibrosis transmembrane conductance regulator modulator profoundly transforms the lived experiences of South African PLwCF providing compelling evidence to advocate for equitable access in resource-limited settings.

**Clinical Implications:**

National funding and rollout of CFTRm for all eligible PLwCF in South Africa are urgently needed, together with interdisciplinary support to navigate modifications to existing care regimens.

## Introduction

Management of cystic fibrosis (CF) has advanced significantly in the past four decades, particularly in high-income settings, with improved life expectancy and clinical outcomes (Felipe Montiel et al. 2024). The major advance in CF care has been the introduction of cystic fibrosis transmembrane conductance regulator modulator (CFTRm) therapy, which has transformed the lives and clinical outcomes of many adults and children living with CF (Bacalhau et al. 2023; Bessonova et al. 2018; King et al. 2022). However, socio-political and regulatory challenges have delayed access to CFTRm in South Africa and other resource-limited settings, where CFTRm treatment is still limited to a relatively small group of patients with eligible mutations who can afford the medication and/or medical insurance (Zampoli et al. 2023a, 2025a).

Cystic fibrosis transmembrane conductance regulator modulator eligibility is determined by an individual’s specific CFTR gene mutation. The triple combination elexacaftor and tezacaftor and ivacaftor (ETI; marketed as Trikafta, Kaftrio, Caftrio or the generic Trixicar) is effective in approximately 85% – 90% of people with CF who carry at least one copy of the most common, F508del, mutation (Bacalhau et al. 2023). A proportion of people with CF who carry relatively rare mutations are not currently amenable to available modulators and remain ineligible for ETI. In South Africa, this includes those who are homozygous for the 3120+1G>A mutation, which is the most common CF-causing mutation in Black African populations (Padoa et al. 1999).

South Africa’s two-tiered healthcare system comprises a well-resourced private sector, serving approximately 16% of the population with medical insurance, and an under-resourced public sector serving the remaining 84%. The list price of branded ETI exceeds R5 million per patient per year, placing it entirely beyond the reach of most South Africans without comprehensive medical insurance or philanthropic support; even generic formulations sourced through alternative channels cost up to R300 000.00 per year, illustrating the profound magnitude of the access barrier.

By the end of December 2023, when this research study was conceptualised, only 77 of the 505 people known to be diagnosed with CF, and approximately 18% of eligible individuals, were receiving CFTRm therapy in South Africa (Zampoli et al. 2023c). At the time of our study, CFTRm was available primarily through private payment or medical insurance, creating profound access inequities (Zampoli et al. 2025a).

While clinical efficacy is well-documented (Edwards et al. 2025; Graeber et al. 2022; Griese et al. 2021; Heijerman et al. 2019; Middleton et al. 2019), including emerging data from South Africa (Zampoli et al. 2023b, 2025b), patient-reported outcomes regarding daily functioning and participation remain underexplored, particularly in resource-limited settings. Specifically, it is not clear how CFTRm influences the lived experience of respiratory symptom and treatment burden, physical activity capacity, social participation and general health and well-being – domains that are central to the World Health Organization’s International Classification of Functioning, Disability and Health (ICF) framework (Bagci et al. 2023; Gomes et al. 2019; Mandrusiak, MacDonald & Watter 2009). Understanding patient-reported, meaningful outcomes is important in the context of South Africa’s stark health inequities (Zampoli et al. 2025a), as evidence may strengthen advocacy for broader, equitable access to CFTRm in South Africa and other resource-limited contexts.

The aim of this research was therefore to describe and explore the lived experience of South African people living with cystic fibrosis (PLwCF) following initiation of CFTRm therapy, with specific objectives to describe their perceptions of change in respiratory symptoms and respiratory treatment burden, physical activity levels and participation, and overall health and well-being.

## Research methods and design

### Study design

A mixed-methods approach was used for this cross-sectional descriptive study, incorporating a quantitative online survey and qualitative data from online semi-structured interviews, using a convergent design for integration.

### Setting

Our study was conducted within the South African CF community. At the time of our study, CFTRm was available exclusively through the private healthcare sector, accessible only to those with comprehensive medical insurance or the financial means to pay out of pocket (Zampoli et al. 2025a). Participants were recruited nationally through the South African Cystic Fibrosis Association (SACFA) network and affiliated CF clinics.

### Study population and sampling strategy

All PLwCF in South Africa, aged > 6 years, who had initiated and were taking any form of CFTRm, irrespective of dose, were eligible to participate, provided they had internet access for online survey or interview participation. Although CFTRm is now licensed for younger children from 2 years of age, at the time of study development, it was available only for children from the age of 6 years in South Africa. Detailed demographic data (biological sex, geographic location, specific CFTR mutation, specific CFTRm formulation) were deliberately not collected for ethical reasons, as the combination of such variables in this small, identifiable population (*n* = 77 nationally) would pose a meaningful risk of re-identification of individual participants. This decision was endorsed by the institutional Ethics Committee. Collection of the age of the person with CF and duration of CFTRm use was considered sufficient for study purposes while protecting participant anonymity, consistent with established practice in rare disease research.

At the end of 2023, 77 PLwCF in South Africa were receiving CFTRm (Zampoli et al. 2023c). We pragmatically aimed to enrol a convenience sample of 20–30 individuals for the survey component to achieve adequate descriptive statistics while acknowledging the constrained population size. For the qualitative component, data collection continued until thematic saturation was achieved. Saturation was assessed through ongoing analysis after each interview, with the research team convening after interviews 3, 4, 5 and 6 to discuss emerging themes. Saturation was determined when: (1) no new themes emerged from the data; (2) existing themes were well-developed with sufficient supporting examples; and (3) the team reached consensus that further interviews were unlikely to substantially change the thematic structure. Saturation was achieved after six interviews, consistent with established guidelines for qualitative interview studies (Malterud, Siersma & Guassora 2016).

For the survey, a convenience sample was recruited through multiple channels: distribution through the SACFA network via email and social media platforms (from 15 February 2025 to end of May 2025); invitation through adult and paediatric CF clinics within the SACFA network; and snowball sampling through CF social networks. For the qualitative component, participants were purposively recruited from the SACFA network, aiming for a balance of age (adults and children), biological sex, geographical location and duration of CFTRm therapy.

### Data collection instruments

A self-designed survey questionnaire was developed for online completion using Google Forms, designed for both self-completion by children aged 12–18 years and adults, and proxy completion by parents of children under 12 years of age. The survey was structured in three parts (Online Appendix 1):

- Part 1: Demographic information: age of the person with CF, whether the respondent was the person with CF or a parent completing on behalf of a child, and duration of CFTRm use.
- Part 2: Recalled experiences during the year prior to starting CFTRm: frequency, severity and impact of respiratory symptoms; perceived burden of respiratory therapy (airway clearance and inhalation therapy); and type, frequency, level and tolerance of physical activity and social participation.
- Part 3: The same questions as in Part 2, with the recall period relating to current experience while taking CFTRm.

Both closed-ended binary (yes or no) and Likert-scale response options were included, with open-ended free-text questions allowing additional detail. The survey underwent content validation by three experts in CF and was pilot tested on three participants (one adult, one adolescent aged 16 years, one parent of a child with CF), whose results were excluded from the final analysis. Minor wording and formatting adjustments were made following piloting. Pilot participants were re-invited to complete the revised questionnaire anonymously; exclusion of pilot responses is a standard precautionary practice to avoid potential inflation of findings from individuals who contributed to instrument refinement, even where changes were minor, and to protect anonymity and confidentiality of these identified individuals.

A qualitative interview guide was collaboratively developed by the research team, based on our study objectives and a literature review. Initial questions were broad and open-ended (e.g. ‘Please describe your experience with CF and how it has affected your life’), followed by focused probes addressing specific domains related to CFTRm therapy (respiratory symptoms, treatment burden, physical activity, participation and well-being). The guide remained flexible, allowing participants to raise unanticipated topics.

### Procedure

Adults living with CF completed the questionnaire themselves; parents completed the form on behalf of children unable to do so independently or assisted their child where necessary. The survey took approximately 30 min to complete. Interviews were conducted with adults living with CF and with parents of children under the age of 16 years, with the option of including the child as well. Interviews lasted approximately 30 min, were conducted online with cameras enabled to allow observation of non-verbal communication and were transcribed verbatim using Microsoft Teams’ built-in artificial intelligence (AI) transcription tool, with complete human verification by the research team.

### Data analysis

Quantitative survey data were exported from Google Forms to Microsoft Excel for cleaning, then imported into IBM^®^ Statistical Package for the Social Sciences (SPSS) (IBM Corporation 2025, United States [US] version 30.0.0.0). The continuous variable, age, was summarised using medians and interquartile ranges (IQRs); categorical variables were summarised using frequencies and percentages. Changes in outcomes before and after CFTRm were analysed using Chi-square tests for categorical variables; Fisher’s exact tests were used when expected cell counts were < 5. Statistical significance was set at *p* < 0.05 (two-tailed). Available case analysis (pairwise deletion) was used for missing data, with denominators varying by item. Age was the only continuous variable in the dataset; median and interquartile range (IQR) were used as the age distribution was not assumed to be normal given the wide range (6–53 years) and relatively small sample. All other variables were categorical or ordinal. Ordinal Likert-scale data were treated as categorical, which was considered appropriate given the response formats used and the sample size.

Interview transcripts were verified against original recordings before analysis. The qualitative analytical approach was descriptive, within a pragmatic research paradigm, adopting a realist and essentialist epistemological stance. Qualitative data from both interviews and open-ended survey responses were analysed together using inductive thematic analysis, following Braun and Clarke’s (2006) six-phase approach: (1) familiarisation with data; (2) generating initial codes; (3) searching for themes; (4) reviewing themes; (5) defining and naming themes and (6) producing the report.

Primary independent coding was conducted by three student researchers (Sinead Thistlewhite, Carese Abrahams and Dalziel Kennedy), with regular input from Brenda M. Morrow and consensus discussions involving all authors. Preliminary themes were reviewed, refined, regrouped and named by the senior investigator (Brenda M. Morrow), and subsequently returned to the full research group for confirmation and final checking. Following separate analyses, quantitative and qualitative findings were integrated using a convergent design, with quantitative results providing evidence of the magnitude and prevalence of changes and qualitative themes illuminating the meaning and lived experience of those changes.

### Researcher reflexivity

The primary investigator (Brenda M. Morrow) is a female PhD-prepared physiotherapist with 30 years’ experience working with people with CF; the last author (Marco Zampoli) is a male PhD-prepared physician with more than a decade’s experience in CF care; Janine Verstraete is a female PhD-prepared physiotherapist. All three are active advocates for CFTRm access in South Africa, creating potential for confirmation bias. To mitigate this, student researchers (Sinead Thistlewhite, Carese Abrahams, Teboho N. Nkosi, Tyra Donnelly and Dalziel Kennedy) conducted all interviews and initial coding; the research team actively sought disconfirming evidence during analysis; and the anonymous survey provided an independent data source less susceptible to social desirability bias.

### Rigour and trustworthiness

Credibility was strengthened through methodological and investigator triangulation. Dependability was ensured through comprehensive audit trails documenting recordings, transcripts, coding evolution and analytical decisions. Confirmability was maintained through transparent reporting grounded in direct participant quotations. Transferability is supported through thick description of the South African CF context, participant demographics and purposive sampling for diversity.

### Ethical considerations

Ethical clearance to conduct our study was obtained from the Faculty of Health Sciences Human Research Ethics Committee (HREC) of the University of Cape Town (No. 851/2024). All procedures were conducted in accordance with the ethical standards of the institutional research committee and with the 1964 Helsinki Declaration and its later amendments. Written informed consent was obtained from all adult participants and from parents or legal guardians of minor participants prior to enrolment; written assent was obtained from child participants where applicable. All interview data were de-identified prior to thematic analysis, and no identifiers were collected in the survey. All study data were stored on password-protected institutional servers accessible only to the research team. Interview recordings were securely deleted after transcription and verification. Survey data were managed through Google Forms with restricted access. Data will be retained for five years in accordance with the University of Cape Town’s research data management policies, after which it will be securely destroyed.

## Results

### Quantitative survey sub-study

#### Participants

Of the estimated 77 individuals taking CFTRm in South Africa at the time of study initiation, 37 completed surveys (response rate: 48%), giving a margin of error of approximately 12% at a 95% confidence interval. The median (IQR) age of participants responding to the survey was 21.5 (16.25–33) years (range: 6–53 years). The majority (*n* = 17; 45.9%) had started CFTRm more than 1 year previously; 15 (40.5%) had started treatment 6 months previously; and five (13.5%) had started within the previous 3 months. Some participants did not answer all survey questions; no pattern was identified in the missing responses. All ages reported are those of the person with CF; where a parent completed the survey on behalf of a child, the child’s age (not the parent’s) was sought and recorded.

#### Respiratory symptoms

**FIGURE 1:**
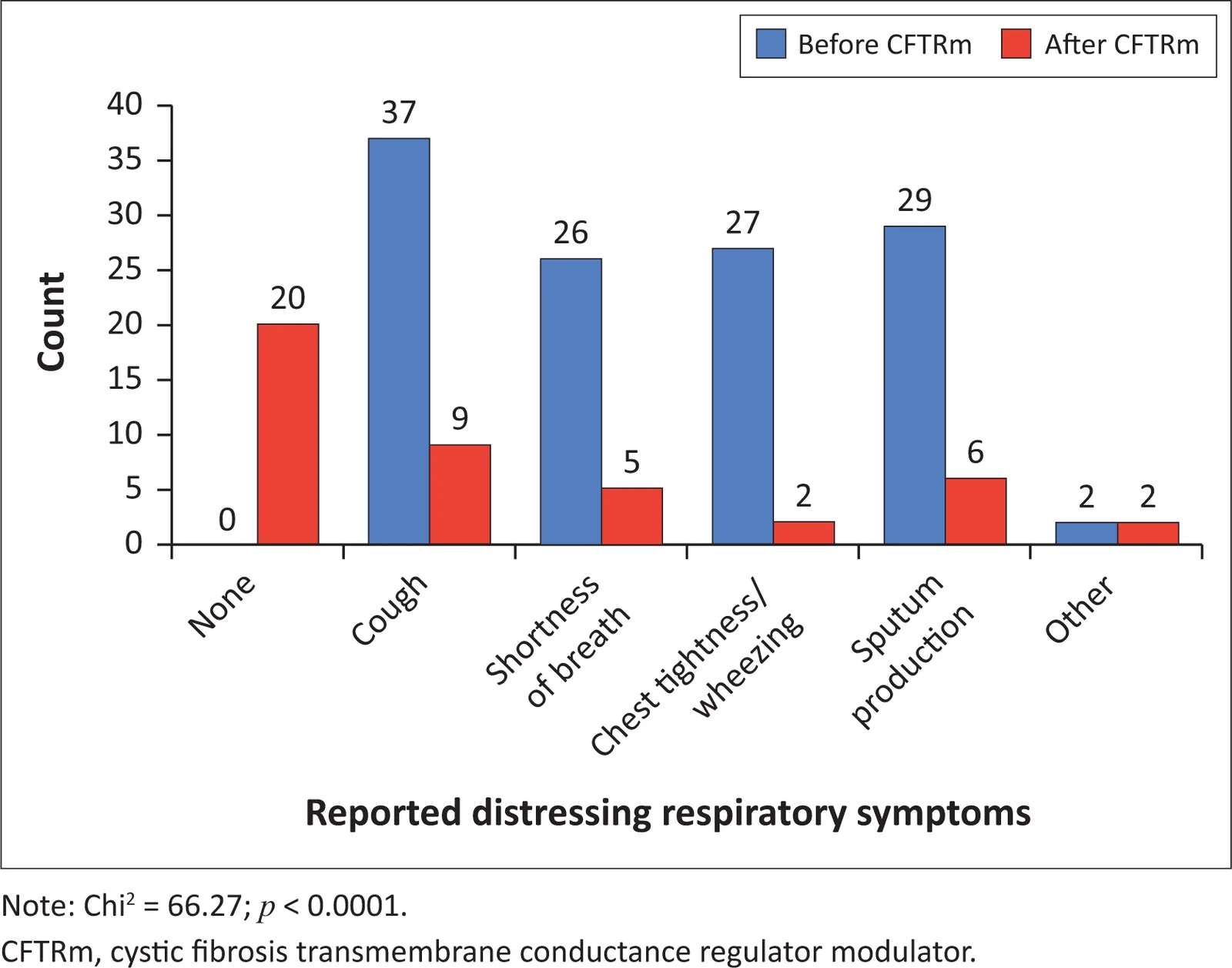
Reported distressing respiratory symptoms before and after starting cystic fibrosis transmembrane conductance regulator modulator therapy (some participants reported more than one symptom).

Participants reported significant improvements in the frequency, nature and severity of respiratory symptoms following CFTRm initiation (Table 1; Figure 1). Before CFTRm, 22 participants (59.5%) reported severe or very severe respiratory symptoms; after CFTRm, 27 (84.4%) reported only very mild symptoms, and none reported severe or very severe symptom severity.

**TABLE 1:** Self-reported changes in respiratory symptoms, burden of respiratory therapy, school or work attendance and participation in recreational activities before and after cystic fibrosis transmembrane conductance regulator modulator initiation.

Domain	Before CFTRm		After CFTRm		*p*-value
	*n*	%	*n*	%	
**Perceived severity of distressing or unpleasant respiratory symptoms (*N* = 37 before; *n* = 32 after)**	-	-	-	-	< 0.0001
Very mild	1	2.7	27	84.4	-
Mild	4	10.8	2	6.3	-
Moderate	10	27.0	3	9.4	-
Severe	12	32.4	0	0.0	-
Very severe	10	27.0	0	0.0	-
**Frequency of any distressing or unpleasant respiratory symptoms (*N* = 37 before; *n* = 36 after)**	-	-	-	-	< 0.0001
Daily	27	73.0	2	5.6	-
Weekly	4	10.8	2	5.6	-
Monthly	3	8.1	1	2.8	-
Rarely	3	8.1	32	88.9	-
**Average reported time spent daily on inhalation and airway clearance therapy (*N* = 37 both)**	-	-	-	-	< 0.0001
More than 3 h	1	2.7	0	0.0	-
2–3 h	7	18.9	0	0.0	-
1–2 h	19	51.4	5	13.5	-
Less than 1 h	10	27.0	32	86.5	-
**Perceived burden of respiratory therapy (*N* = 37 both) [1 = not at all burdensome; 5 = extremely burdensome]**	-	-	-	-	< 0.0001
1 (not at all burdensome)	1	2.7	25	67.6	-
2	1	2.7	7	18.9	-
3	6	16.2	4	10.8	-
4	14	37.8	1	2.7	-
5 (extremely burdensome)	15	40.5	0	0.0	-
**Reported frequency of missed school/workdays owing to CF (*N* = 37 both)**	-	-	-	-	< 0.0001
Never or rarely	2	5.4	27	73.0	-
One or more day every month	11	29.7	1	2.7	-
One or more week every 6 months	13	35.1	3	8.1	-
One or more week every year	5	13.5	5	13.5	-
Other	6	16.2	1	2.7	-
**Reported frequency of recreational activities (*N* = 37 both) [1 = never; 5 = very often]**	-	-	-	-	0.006
1 (never)	4	10.8	1	2.7	-
2	9	24.3	1	2.7	-
3	10	27.0	6	16.2	-
4	6	16.2	11	29.7	-
5 (very often)	8	21.6	18	48.6	-

CFTRm, cystic fibrosis transmembrane conductance regulator modulator; CF, cystic fibrosis.

#### Respiratory therapy

**FIGURE 2:**
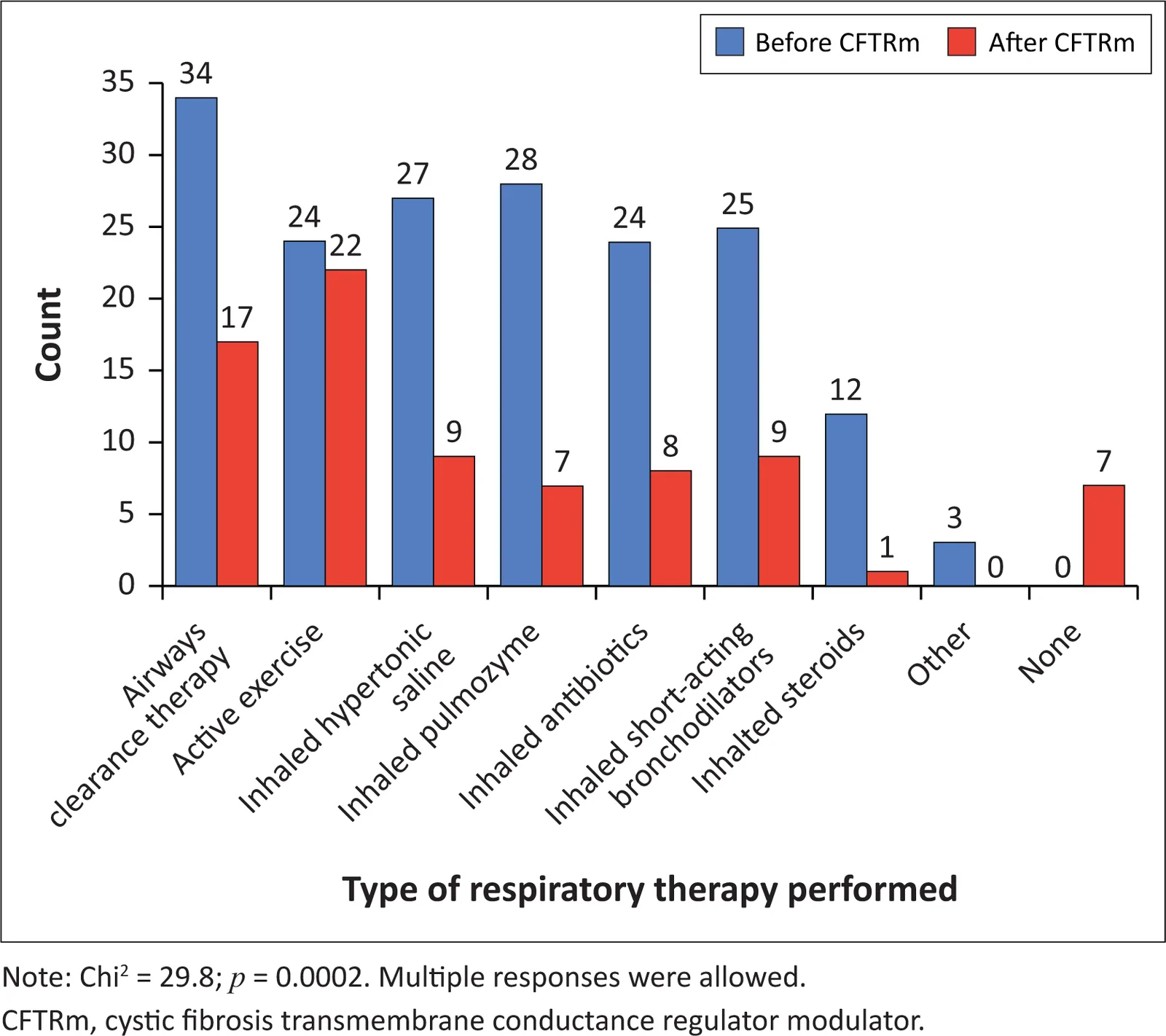
Type of respiratory therapy performed before and after starting cystic fibrosis transmembrane conductance regulator modulator therapy.

The perceived frequency, duration and burden of respiratory therapy changed significantly following CFTRm initiation (Table 1; Figure 2). Before CFTRm, 15 participants (40.5%) rated respiratory therapy as extremely burdensome (Likert-scale 5/5), with only one participant (2.7%) rating it as not at all burdensome (1/5). After CFTRm, none rated it as extremely burdensome, and most (*n* = 25; 67.6%) perceived it as not burdensome at all. All modalities of respiratory therapy were reduced after CFTRm initiation, with five participants (13.5%) no longer performing any form of respiratory therapy.

#### Physical activity

**FIGURE 3:**
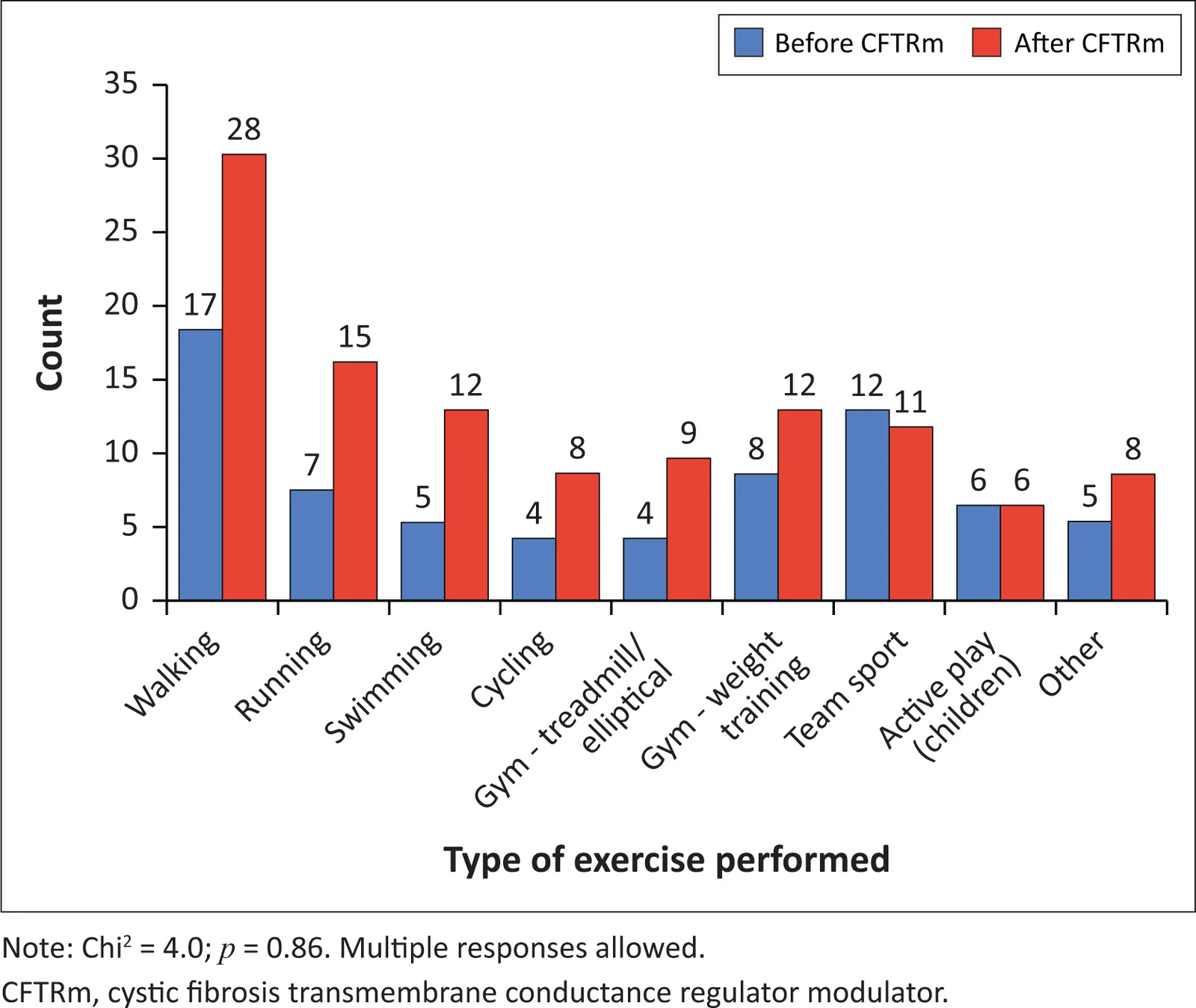
Types of exercise performed before (*n* = 23) and after (*n* = 34) starting cystic fibrosis transmembrane conductance regulator modulator therapy.

Before starting CFTRm, 23 respondents (62.2%) reported regular exercise participation. Reported barriers to exercise pre-CFTRm included an increase in unpleasant symptoms when exercising (*n* = 13, 92.9%), being too ill (*n* = 11, 78.6%), poor lung function (*n* = 5, 35.7%), and anxiety, fear of exercise or lack of enjoyment (*n* = 3 each). Of those who exercised, the most common activities were walking (*n* = 17; 73.9%) and team sports (*n* = 12; 52.2%) (Figure 3).

After starting CFTRm, there was a significant increase in regular exercise participation, with the majority (*n* = 34, 91.9%) engaging in one or more forms of exercise (*p* = 0.04). The most common activities were walking (*n* = 28; 82.5%), running (*n* = 15; 44.1%), swimming (*n* = 12; 35.3%) and weight training (*n* = 12; 35.3%); however, there was no significant difference in the type of exercise performed before versus after CFTRm (*p* = 0.86; Figure 3). Three participants reported not exercising after CFTRm initiation, citing lack of enjoyment, continued symptoms in established severe CF-related lung disease (*n* = 2) and difficulty establishing an exercise routine (*n* = 1). Exercise session duration and frequency did not change significantly (*p* = 0.5 and *p* = 0.8, respectively).

#### Participation and daily living

**FIGURE 4:**
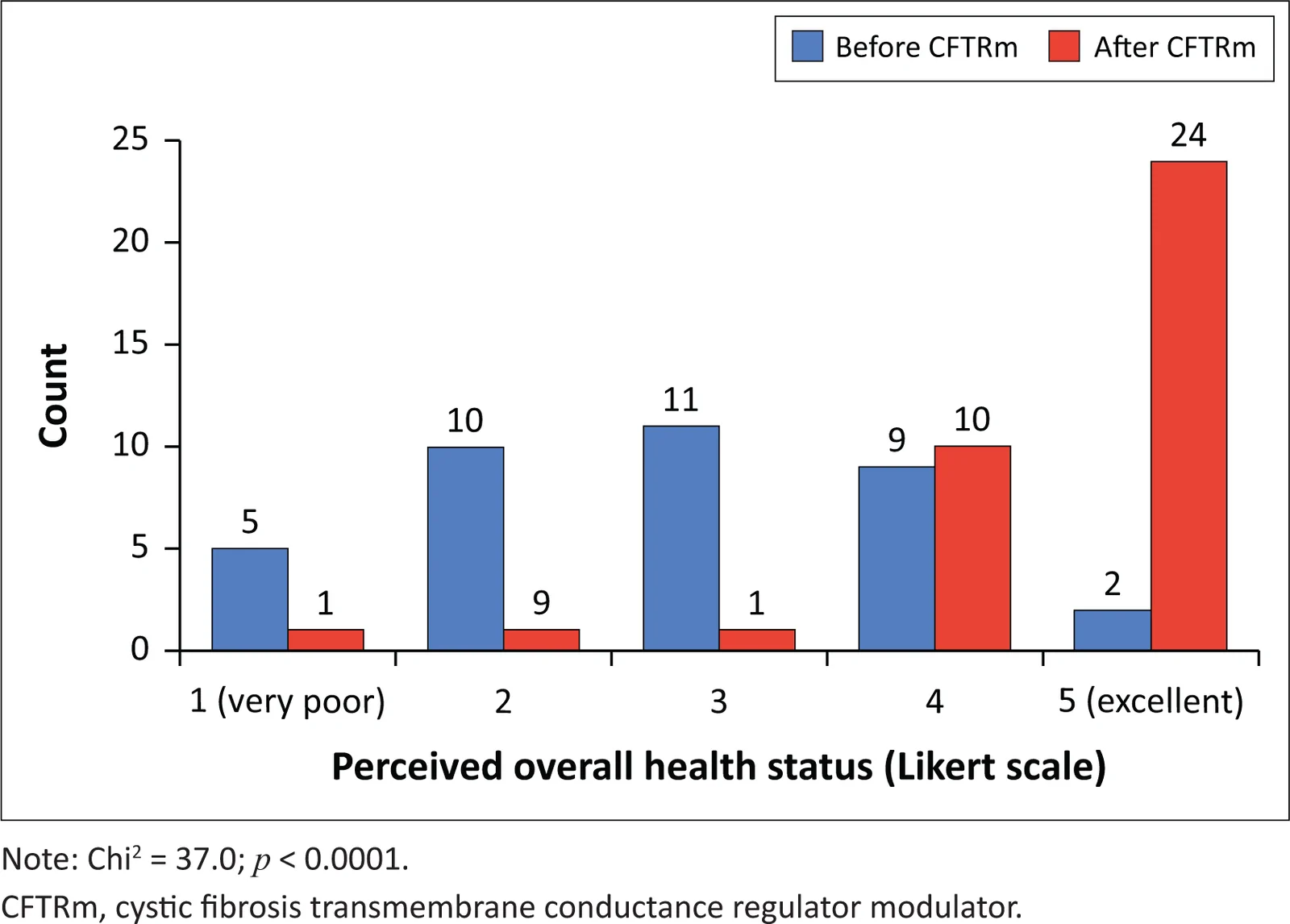
Perceived health status before and after starting cystic fibrosis transmembrane conductance regulator modulator.

There was a significant improvement in school and work attendance (Table 1; *p* < 0.0001). The proportion of participants who reported never or rarely missing school or work increased from 5.4% (*n* = 2) before CFTRm to 73.0% (*n* = 27) after CFTRm. Frequent engagement in recreational activities with friends or peers increased from 21.6% (*n* = 8) before CFTRm to 48.6% (*n* = 18) after CFTRm (*p* = 0.006). Perceived excellent health increased from 5.4% (*n* = 2) to 64.9% (*n* = 24) (Figure 4; *p* < 0.0001).

### Qualitative sub-study

Six participants were selected through purposive sampling: three adults with CF aged 19 years, 23 years and 40 years (all diagnosed in childhood), and three parents of children with CF aged 7–9 years. Participants had been taking CFTRm for between 6 months and 4 years. This diversity in age, perspective (self-report versus parent proxy) and duration of CFTRm experience ensured the capture of varied experiences across the CFTRm-using population.

Data saturation was achieved after six interviews, in combination with open-ended survey responses, as evidenced by thematic consistency across participants, the absence of new themes in later interviews and convergence of findings despite the intended sample heterogeneity.

Thematic analysis revealed 10 major themes describing the experiences of South African PLwCF after starting CFTRm (Table 2). Themes were consistent across gender, age (adults versus children) and duration of CFTRm use:

1. **Theme 1 – Transformation of respiratory health** encompassed two subthemes: (1.1) *dramatic reduction in respiratory symptoms* and (1.2) *improved physiological indicators*. Participants reported dramatic improvements in cough, mucus production and ease of breathing, alongside objective improvements in lung function and sweat chloride. ‘I can laugh again without coughing’ (Survey respondent 19).

2. **Theme 2 – Dramatic reduction in respiratory treatment burden** comprised two subthemes: (2.1) *saving time* and (2.2) *simplified regimens*. Participants described spending substantially less time on airway clearance and inhalation therapy, with marked simplification of daily regimens:

‘It saved us about 3 to 4 hours a day of treatments … we are actually at the point now where she has no physio or nebulising outside of when she’s sick.’ (Parent of child 1, female, aged 7 years)

**TABLE 2:** Major themes, subthemes and illustrative quotes.

Theme	Subtheme	Illustrative quote	Participant
**1. Transformation of respiratory health**	1.1. Dramatic reduction in respiratory symptoms	‘… the ease of the cough is just incredible. You’re not coughing for 20 or 30 seconds feeling like you’re going to pass out and hurl. You only cough like normal people do, and it’s over.’	Adult 1 (female, aged 40 years)
		‘The first thing we noticed … it’s too quiet in the house. We are used to [*child name*] every night … there’s always a cough … Now there is no cough … For us as parents, that’s the biggest gift that you can give us, is a child without a cough.’	Parent of child 3 (male, aged 9 years)
		‘We can barely get stuff out [*mucous*] … there’s no gunk in her lungs … we can barely get a sample.’	Parent of child 1 (female, aged 7 years)
		‘I can sleep full nights without waking up in the middle of the night coughing.’	Survey respondent 30
	1.2. Improved physiological indicators	‘Within five weeks … The sweat test came down to 28 [*from high 90s/hundreds*] … her body’s technically functioning normally.’	Parent of child 1 (female, aged 7 years)
		‘My lung function has gone up to 96%!! The highest it has been in over 11 years!’	Survey respondent 32
**2. Dramatic reduction in treatment burden**	2.1. Saving time	‘I had to do 20, 30 minutes of physio … I do only nine minutes now … And my chest is clear.’	Adult 1 (female, aged 40 years)
		‘I only do inhaled Colimycin twice a day … less than an hour. Previous treatments took 2–3 hours.’	Survey respondent 36
	2.2. Simplified regimens	‘He was on Bactrim 3 times a week, but we have stopped that. The Tobi we stopped completely. The Pulmozyme we do on weekends if we remember … Dr [*name*] said we can just give it a break.’	Parent of child 3 (male, aged 9 years)
		‘The real change to my life has been my long daily checklist of things to do has been cut in half. I no longer have to nebulise once or twice a day. The number of tablets I need to take has halved and I seldom need my inhalers.’	Survey respondent 18
**3. Liberation and normalisation of daily life**	3.1. Freedom from disease constraints	‘We can go outside, and he can experience small things that other kids don’t even notice … One night we went outside, and he said, “wow Mom it’s so nice to see the stars. It’s so nice to be outside and enjoy the outside life,” because he is not used to it.’	Parent of child 3 (male, aged 9 years)
		‘We moved him from a private school to a normal, local government school because now he can be in a class of 30–35 kids and not get sick. Before Trikafta, he had to be in a class that is 15 or max 20 kids.’	Parent of child 3 (male aged 9 years)
	3.2. Living like a ‘normal’ person	‘It’s the closest to having a normal child. She goes to school in a normal way … it’s as if she’s living a normal life … literally has gone from a child, you’d have to sanitise hands. We would never go to [*a restaurant*] indoors play area with her ever. To someone who can actually do everything now that any other child can do.’	Parent of child 1 (female, aged 7 years)
		‘Since being on Trikafta I regularly forget the effects CF has on me.’	Survey respondent 22
**4. Enhanced physical capacity and participation**	4.1. Improved exercise tolerance and athletic performance	‘He’s got extra athletics at night-time now. We couldn’t do it before the Trikafta ….’	Parent of child 3 (male, aged 9 years)
		‘For our trials … I would have to run two to five k’s … went from running like an eight minute to a six forty-five [*2K time*] … the general ability to breathe throughout the running was a lot smoother.’	Adult 2 (male, aged 19 years)
	4.2. Increased social and recreational participation	‘When everybody’s playing outside at nighttime … he can’t go outside because he gets sick. That was the worst for me as a mom, it broke my heart … He could never go on a rugby tour … He went to a rugby tour a few months ago … He didn’t get sick.’	Parent of child 3, (male, aged 9 years)
**5. Profound psychosocial and emotional impact**	5.1. Relief from anxiety and emotional burden	‘Before I started CFTR modulators, I was listed for a transplant … my treating team actually tell me that they were very worried. They estimated that I had about two months left to live.’	Adult 1 (female, aged 40 years)
		‘Each time we go, we go with certain fears … then when you see, “ok there’s weight gain or the lung function test is good” you go relieved but tired … with Trikafta, there’s definitely been relief.’	Parent of child 2 (female, aged 8 years)
		‘The improvement in my quality of life is so great that it is hard to quantify.’	Survey respondent 8
	5.2. Fear of change in treatment regimen	‘I didn’t want to stop [*physiotherapy*] because it was the only thing I could control.’	Parent of child 1 (female, aged 7 years)
		5.3. Hope and future possibilities	‘These drugs are truly life changing. Taking me from my death bed to being able to live a “normal life … being about to enjoy time with family and friends and actually plan some sort of future.”’	Survey respondent 10
		‘I can honestly say that Trikafta has given me a whole new meaning on living and enjoying every experience of life there is to discover.’	Survey respondent 20
		‘I have been put on hold on the transplant list. I can carry on with these puppies for the next couple of years!’ [*referring to the participant’s lungs*]	Survey respondent 26
**6. Improved growth and nutritional status**	-	‘Since [*child name*] has been taking the Trifakta there has been an enormous … weight gain and growth ….’	Parent of child 2 (female, aged 8 years)
**7. Reduction in hospitalisations and acute Illnesses**	-	‘Since she’s on the Trikafta, she has not been admitted to hospital. She started in September. We’ve not needed to give her any antibiotics for anything.’	Parent of child 2 (female, aged 8 years)
**8. Family impact and adaptation**	8.1. Changed family dynamics	‘Husband had to do way more than the normal father does in a household for the children … the impact of CF on our family has been a journey.’	Adult 1 (female, aged 40 years)
		‘There was a lot of fighting and crying.’ [*before CFTRm*]	Parent of child 1 (female, aged 7 years)
	8.2. Financial and practical challenges	‘We were able to raise funds to buy a box of Trixacar for 75,000 Rand in Argentina … [*Medical Aid*] pays half of it, and there’s a charity that pays the other half. So that’s the only way we’ve been able to gain access.’	Parent of child 1 (female, aged 7 years)
		‘Before the Trikafta I used a lot of Pediasure, and that is quite expensive … But now I don’t have to buy that much Pediasure … Its saving you a little bit.’	Parent of child 3 (male, aged 9 years)
**9. Increased energy and reduced fatigue**	-	‘After starting Trikafta I have noticed a huge change in energy levels and doing my daily routine and tasks like uni’ is so much easier.’	Survey respondent 30
**10. Altruism and hope**	-	‘I wish I could give it to everybody and tell them there’s nothing better in life than that. Because really, it is life changing. It is not just something you can take- it is life changing.’	Parent of child 3 (male, aged 9 years)
		‘People need this [*Trikafta*] … we need to get it to everyone.’	Adult 3 (male, aged 23 years)
		‘I wish that one day, every single CF patient all over the World will be able to have access to this medication.’	Survey Respondent 32

This theme is closely interlinked with Theme 3: reduced treatment burden was a central driver of the liberation and normalisation of daily life that participants described.

3. **Theme 3 – Liberation and normalisation of daily life** included two subthemes: (3.1) *freedom from disease constraints* and (3.2) *living like a ‘normal’ person*. Participants expressed a profound sense of freedom from physical limitations that had previously dominated their daily lives:

‘The biggest change I’ve noticed is how easy it is to just live life now … Now if I’ve got my Trikafta [*ETI*] with me I’m fine. I can just live life.’ (Adult 3, male, aged 23 years)

4. **Theme 4 – Enhanced physical capacity and participation** encompassed two subthemes: (4.1) *improved exercise tolerance and athletic performance* and (4.2) *increased social and recreational participation*. Participants described improvements in exercise capacity and engagement in activities previously precluded by symptoms:

‘In February this year, I ran my first half marathon, a 21K race and 12 months before that I could barely run a 3K without stopping … I ran the whole 21 … didn’t stop. Like I didn’t walk.’ (Adult 3, male, aged 23 years)

Theme 4 is meaningfully interlinked with Theme 3, as increased physical capacity directly enabled the normalisation of daily life participants described, and with Theme 9, as improved energy and reduced fatigue underpinned these physical gains.

5. **Theme 5 – Profound psychosocial and emotional impact** comprised three subthemes: (5.1) *relief from anxiety and emotional burden*, (5.2) *fear of change in treatment regimen*, and (5.3) *hope and future possibilities*. While most participants described profound relief from the emotional burden of symptomatic CF, some described anxiety associated with reducing long-established treatment routines:

‘I think when you’re 43 and you’re used to having a nebuliser and physio your whole life, twice a day, you kind of get panicky when you don’t do that.’ (Adult 1, female, aged 40 years)

This theme is interlinked with Theme 2, as the very reduction in treatment burden that participants welcomed also generated psychological discomfort for some, and with Theme 8, where financial anxiety around sustaining CFTRm access emerged as a new source of psychosocial burden.

6. **Theme 6 – Improved growth and nutritional status.** Adult and parents of paediatric participants described marked improvements in weight gain and growth trajectories. ‘Obviously the biggest change I’ve seen is the weight gain. I’ve been able to put on weight more consistently and healthily’ (Adult 2, male, aged 19 years).

7. **Theme 7 – Reduction in hospitalisations and acute illnesses.** Participants reported substantially fewer hospital admissions and reduced antibiotic requirements following CFTRm initiation. ‘She was in hospital … in and out two weeks at a time, probably for three months … She hasn’t been in hospital since [*starting Trikafta*]’ (Parent of child 1, female, aged 7 years).

8. **Theme 8 – Family impact and adaptation** included two subthemes: (8.1) *changed family dynamics* and (8.2) *financial and practical challenges*. Cystic fibrosis transmembrane conductance regulator modulator profoundly affected the wider family unit, easing caregiver burden while simultaneously raising financial pressures related to the very high cost of therapy. ‘The price as it is now $326 000.00 per year for the medication … unattainable …’ (Parent of child 1, female, aged 7 years). This theme is interlinked with Theme 5, as financial anxiety around sustaining access to CFTRm emerged as a new source of psychosocial burden even as other anxieties resolved.

9. **Theme 9 – Increased energy and reduced fatigue.** Participants described improved energy levels and stamina, enabling greater daily function and participation. ‘Energy in general, I don’t feel as tired or lethargic. I’m able to get more done. I’m able to do my sports more often without getting tired’ (Adult 2; male, aged 19 years). Theme 9 is interlinked with Theme 4, as improved energy directly underpinned the physical capacity gains and expanded participation participants reported.

10. **Theme 10 – Altruism and hope.** Participants expressed a wish for equitable CFTRm access for all PLwCF, reflecting both gratitude for their own access and acute awareness of those unable to access treatment. ‘And that is one of the reasons why we are working so hard and advocating … I just want patients to have a chance at normal life’ (Adult 1, female, aged 40 years).

Illustrative quotes from interview participants and selected free-text survey responses are presented for each theme in Table 2. Brand names appearing in participant quotes (e.g. Trikafta, Kaftrio, Caftrio, Trixicar) are all commercial formulations of ETI.

## Discussion

This mixed-methods study provides compelling preliminary evidence of the positive lived experiences of PLwCF in South Africa who have been able to access CFTRm therapy. The integrated quantitative and qualitative results present a credible and coherent picture of improved well-being following CFTRm initiation, with substantially reduced respiratory symptoms and treatment burden, improved participation in recreational and physical activities and reduced school or work absenteeism, along with new hope and optimism for the future. No major discordance between the quantitative and qualitative components was identified.

Participants recalled many distressing symptoms prior to starting CFTRm, including breathing difficulties, cough, malnutrition, haemoptysis, difficulty clearing the lungs and sleep disruption. These symptoms are well described in CF (Castellani & Assael 2017; Elborn 2016) and are known to have substantial psychosocial effects, with associated impact on participation and both patient and caregiver quality of life (Dobra et al. 2025; Quittner et al. 2014). Following CFTRm initiation, participants in both arms of our study reported significant reductions in the perceived severity and frequency of these symptoms, along with improved lung function and better ability to participate in physical, social and recreational activities. These findings are consistent with multiple studies documenting the effectiveness of CFTRm on pulmonary function, nutrition, pulmonary exacerbations, physical activity and fitness, social participation, psychosocial well-being and health-related quality of life in different settings (Bacalhau et al. 2023; Basile et al. 2024; Berthold et al. 2024; Francesca et al. 2025; Graeber et al. 2022; Heijerman et al. 2019; Middleton et al. 2019; Van Citters et al. 2025).

Respiratory therapy, including airway clearance therapy, has been identified as one of the most burdensome and time-consuming of routine CF treatments (Dziuban et al. 2010; Gifford et al. 2020; Sawicki, Sellers & Robinson 2009), and in our cohort, it constituted a significant perceived barrier to social, work or school and physical participation prior to CFTRm initiation. With improved respiratory symptoms following CFTRm, there was a corresponding decrease in perceived treatment burden in both study components, with all participants reporting reductions in the frequency, complexity and/or duration of respiratory therapy. Our finding that virtually all participants reduced respiratory therapy after CFTRm initiation in this South African cohort differs from findings in high-income settings, where the majority of participants continue established regimens, even without perceived or objective benefit, after starting CFTRm (Basile et al. 2024; Gifford et al. 2020). This difference may reflect a lower threshold for therapy reduction in a severely resource-limited health system and limited access to specialised CF care teams to guide decision-making. This suggestion warrants further study. Reducing therapeutic burden has been identified as a priority for PLwCF (Hisert et al. 2023), yet evidence to guide modification or discontinuation of respiratory therapy following CFTRm remains limited (Terlizzi & Lopes-Pacheco 2025). The SIMPLIFY trial (‘Impact of Discontinuing Chronic Therapies in People with Cystic Fibrosis on Highly Effective CFTR Modulator Therapy’ trial) demonstrated non-inferiority of stopping hypertonic saline or dornase alfa for 6 weeks in those with well-preserved pulmonary function (Mayer-Hamblett et al. 2023), suggesting modification may be appropriate for some patients. The change in treatment burden reported in our study was described not only in terms of clinical treatment ease but also in terms of restored normality, independence and time to participate in daily life activities. Conversely, some participants described the psychological challenge of moving away from long-standing routines, with ongoing anxiety, fear and uncertainty associated with treatment changes. This finding has been similarly reported previously (Gruber et al. 2023). The complexities of psychosocial well-being in the CFTRm era, including new challenges around self-identity, increasing demands of work and school, and ‘survivor guilt’ at being able to access life-saving treatments while others cannot, have been documented previously (Dobra et al. 2025). In the South African context, where CFTRm remains accessible only to those with comprehensive medical insurance, survivor guilt may underlie the many participants’ expressions of hope for universal access. These findings underscore the importance of interdisciplinary clinical support to help patients navigate the psychosocial and practical aspects of CFTRm initiation and safe modification of pre-existing treatment regimens.

Cystic fibrosis affects not only the individual but also their caregivers and families, with depression and anxiety symptoms up to three times more prevalent in PLwCF and caregivers than in the general population (Quittner et al. 2014). Qualitative sub-study participants described the strain on caregivers and family functioning prior to CFTRm initiation, including caregiver burnout and altered family roles – findings consistent with those of Quittner et al. (2014). Following CFTRm initiation, the perceived burden on patients and caregivers eased, with a new optimism and hope for the future, consistent with a qualitative study in adults with CF in which ‘hope’ was the overriding emerging theme and participants described a new ‘stability’ and increased sense of control (Page, Goldenberg & Matthews 2022).

### Limitations

Our study has several limitations. Recall bias is a significant risk, as participants were required to reflect on past experiences. The survey sample was small, and selection bias may have occurred if those with particularly strong experiences were more motivated to participate. Given that CFTRm access in South Africa is relatively recent, responses may reflect a ‘honeymoon period’ effect, as described by Dellon and Prieur (2025), and the absence of adverse events reports may reflect minimisation because of fear of losing access to therapy, or selection bias favouring those with positive experiences (Terlizzi & Lopes-Pacheco 2025). The anonymous nature of the survey precluded the extraction of clinical data and correlation with objective clinical outcomes. Findings have limited generalisability beyond our study population, as participants are skewed towards the higher socio-economic sector. Member checking was not conducted, representing a limitation in confirmability assurance; however, triangulation of findings across multiple data sources strengthens confidence in the themes identified. Future research in larger samples following longer-term, more widespread use of CFTRm is recommended. Furthermore, the direction of recall bias is unlikely to be neutral: participants who have experienced substantial benefit from CFTRm may retrospectively perceive their pre-CFTRm state as worse than they would have recalled it at the true baseline, potentially exaggerating the apparent magnitude of change. This form of response shift or contrast bias, which operates in addition to simple recall bias, may be particularly pronounced among those who have been on CFTRm longest, and cannot be fully mitigated within this retrospective cross-sectional design.

### Policy and research implications

While formal cost-effectiveness analysis was beyond the scope of our study, the reported reductions in hospitalisations, respiratory therapy and work or school absenteeism suggest potential for cost offsets warranting formal economic evaluation in the South African context, although cost-effectiveness of CFTRm has not been clearly demonstrated in other settings (Edwards et al. 2025; Marshall et al. 2023; Sharma et al. 2018; Smith & Barry 2020; Vadagam et al. 2018; Wherry et al. 2020; Whiting et al. 2014). Beyond direct healthcare cost offsets, the observed improvements in patient and caregiver well-being and reduction in caregiver burden may have broader economic implications through increased workforce participation and productivity, dimensions that should be included in any formal health economic evaluation in the South African context.

At the time of writing, at least half the eligible PLwCF in South Africa remain unable to access CFTRm (Zampoli et al. 2025a), and are still reliant on traditional, burdensome management strategies with associated substantial psychosocial impact (Ancel et al. 2022). Without equitable access, the benefits of CFTRm remain confined to a privileged minority, further widening the healthcare gap between rich and poor (Zampoli et al. 2025a). The World Health Organisation’s addition of CFTRm to its essential medicines list in September 2025 highlights the importance of prioritising global access to these therapies. Urgent national rollout and funding of CFTRm for all eligible PLwCF in South Africa, including those in the public sector, are needed to achieve the Sustainable Development Goal of health for all.

## Conclusion

Cystic fibrosis transmembrane conductance regulator modulator therapy has positively transformed the lived experiences of this cohort of South Africans living with CF, with reduced respiratory symptoms and respiratory treatment burden, and improvements in physical activity, social participation and well-being, suggesting improved overall quality of life. These findings demonstrate that the impact of CFTRm extends beyond clinical metrics, encompassing profound improvements in lived experience, participation and family well-being – outcomes central to health equity and quality of life. This evidence strengthens the case for prioritising equitable CFTRm access in South Africa and other resource-limited settings, so that all eligible PLwCF can realise their full potential.
